# Review: Bioengineering strategies to probe T cell mechanobiology

**DOI:** 10.1063/1.5006599

**Published:** 2018-03-29

**Authors:** Adi de la Zerda, Michael J. Kratochvil, Nicholas A. Suhar, Sarah C. Heilshorn

**Affiliations:** 1 Department of Materials Science and Engineering, Stanford University, Stanford, California 94305, USA; 2 Department of Medicine, Division of Infectious Disease, Stanford University, Stanford, California 94305, USA

## Abstract

T cells play a major role in adaptive immune response, and T cell dysfunction can lead to the progression of several diseases that are often associated with changes in the mechanical properties of tissues. However, the concept that mechanical forces play a vital role in T cell activation and signaling is relatively new. The endogenous T cell microenvironment is highly complex and dynamic, involving multiple, simultaneous cell-cell and cell-matrix interactions. This native complexity has made it a challenge to isolate the effects of mechanical stimuli on T cell activation. In response, researchers have begun developing engineered platforms that recapitulate key aspects of the native microenvironment to dissect these complex interactions in order to gain a better understanding of T cell mechanotransduction. In this review, we first describe some of the unique characteristics of T cells and the mounting research that has shown they are mechanosensitive. We then detail the specific bioengineering strategies that have been used to date to measure and perturb the mechanical forces at play during T cell activation. In addition, we look at engineering strategies that have been used successfully in mechanotransduction studies for other cell types and describe adaptations that may make them suitable for use with T cells. These engineering strategies can be classified as 2D, so-called 2.5D, or 3D culture systems. In the future, findings from this emerging field will lead to an optimization of culture environments for T cell expansion and the development of new T cell immunotherapies for cancer and other immune diseases.

## I. INTRODUCTION

In recent years, the field of mechanobiology and how forces influence the behavior of cells and tissues has become an important area of research. Recent data showing a link between mechanical signaling and the pathogenesis of several disorders highlight the significance of understanding how tissue mechanics convert into biochemical signals,^1^ an understanding of which may elucidate a greater knowledge of disease progression. For a number of years, mechanical degradation of tissues was thought to be a symptom of disease. However, now there is a growing shift in the field that instead views abnormalities in tissue mechanics and dysfunctional mechanotransduction as not the end result, but rather significant contributors to disease progression. One example is breast cancer, where it has been shown that an increase in tissue stiffness promotes metastasis *in vitro* and *in vivo* and where there is active research about the use of T cells with improved activity to inhibit this malignancy.^2^ Additionally, several studies have reported that tissue mechanics are significantly altered in inflamed organs. Inflamed organs can result from either injury, infection, or autoimmune reaction,^3^ and since T cells participate in many of these inflammatory responses, T cell mechanobiology has become an intense area of research as well.

**FIG. 1.**
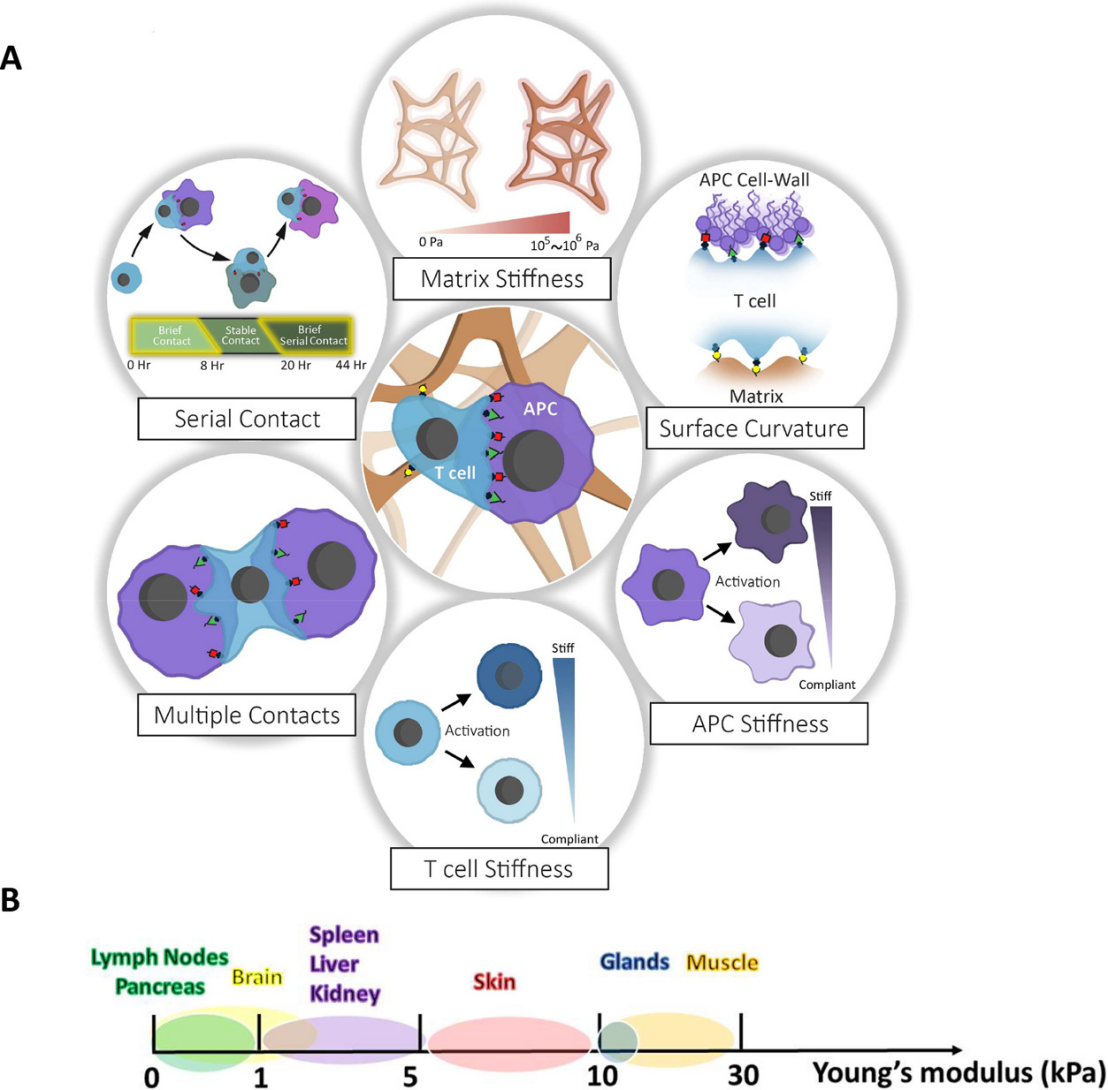
Microenvironmental cues that may impact T cell mechanotransduction. (a) Biophysical/biomechanical factors affecting T cell mechanotransduction. The description begins with the top panel and moves clockwise: T cells encounter a wide range of microenvironments in the body with a diversity of *matrix stiffness* (e.g., Young's modulus, E) values ranging from 10 to 10^6^ Pa and matrix topography that result in cell *surface curvature.* T cells also encounter differences in *APC stiffness*, since the APC membrane rigidity dynamically changes in response to cues from inflammation. The *T cell stiffness* itself is also dynamic, with the membrane rigidity changing during immunological synapse (IS) formation and T cell activation. A single T cell can simultaneously form *multiple contacts* with several APCs. Before forming a stable IS, the T cell can sequentially encounter different APCs for brief periods of time, leading to *serial contact* with cells of differing mechanical properties. (b) Stiffness of common tissues and organs where T cell actions take place. Stiffness is presented in Young's modulus in kPa.^5–7^

T cell function in a highly complex and dynamic mechanical microenvironment in which they undergo cell-cell and cell-matrix interactions, all of which may affect T cell mechanotransduction and the resulting activation responses [Fig. 1(a)]. As T cells circulate throughout the body to locate antigen presenting cells (APCs), they come into contact with differing microenvironments that have varied topography and mechanical stiffness [Fig. 1(b)].^4,5^ Simultaneously, the T cell is processing highly complex interactions with one or more APCs, which also provide multiple independent mechanical stimuli for any one T cell. When a T cell encounters an APC, it forms an immunological synapse (IS) that connects the APC's peptide-major histocompatability complex (pMHC) with the T cell receptor (TCR). At the site of the IS, the T cell changes its morphology to form invadosome-like protrusions that physically push against and probe the membrane of the APC. The T cell's ability to exert force on the APC membrane during this interaction is critical for T cell activation,^8^ as T cells that are unable to exert forces on the APC have a defective activation response.^9^ Another layer of complexity to this interaction is that the APC's membrane rigidity dynamically changes in response to cues from inflammation and the IS,^10,11^ while simultaneously the activated T cell's membrane rigidity also changes and becomes more compliant.^12^ These changes in membrane rigidity may reflect the T cell's ability to sense and respond to fluctuating mechanical cues while simultaneously being activated by the APC. Finally, another dimension to consider is that a single T cell may simultaneously interact with multiple APCs^13^ as well as sequentially encounter different APCs for brief periods of time, both of which bring with it a number of other mechanical stimulants that may affect T cell behavior. As an example of when this may occur, in the case of a pMHC complex having a weak affinity to the TCR, several APC encounters are necessary in order to reach a critical activation threshold.^14^ These latter behaviors in particular, impose a significant challenge to researchers trying to dissect the roles of mechanical cues on T cell activation.

Reductionist approaches try to reduce the complexity of T cell interaction with the microenvironment to enable quantitative biology. Bioengineers employ different strategies to dissect the cellular responses to mechanical cues by creating natural and synthetic platforms that perturb and/or quantify T cell mechanobiology. These strategies have led to the relatively new discovery that T cells are mechanosensitive and the identification of the TCR as a module where force generation can occur.^15–17^

T cells are constantly exerting forces and being submitted to forces, both during their migration and while interacting with their cognate APC. Recently, a study revealed that T cells leverage these mechanical forces to aid in cytotoxic activity against target cells.^9^ Other key results revealed that T cells can modify their growth and proliferation based on different substrate stiffness and substrate topologies.^9,18–21^ Several studies have also suggested that extracellular mechanical forces can facilitate activation of surface receptors^22^ and that pMHC complex recognition may be mediated by mechanical cues from both the APC and the extracellular matrix (ECM),^23^ however to date, the only mechanoreceptor for T cells that has been experimentally validated is the TCR, and the downstream signaling mechanisms remain unknown.^24^ In addition, many findings on how mechanics influence T cell activation are potentially contradictory, which may be attributed to the non-physiological nature of most systems used to date. Overcoming this gap in knowledge will require the efforts of biologists, engineers, and clinicians alike to develop techniques that are physiologically relevant and reductionist to characterize the underlying features in T cells responsible for these mechanotransductive pathways.

Studying these mechanotransduction pathways and the mechanics that affect T cell function within a living host is not just of interest to the scientific community, but signifies a research area that is fundamental to understanding immune response and a necessary step in developing novel therapeutic strategies. Studies report that tissue rigidity changes during the course of disease.^1,3^ For example, most people are familiar with the experience of having a physician touch their lymph nodes to detect the perceived stiffness, as this is often correlated with inflammation and malignancy. In scientific studies, the stiffness of the lymph nodes has been reported to be in the range of 120 Pa to 1 kPa, as detected by a variety of methods including shear-wave ultrasound elastography and a tactile sensor.^3,5,6^ By elucidating how mechanics influence T cell activation, we may be able to identify how to both encourage T cell activation for fighting infections and cancers as well as to suppress T cell activation for controlling autoimmune disease. Local alteration of tissue stiffness by drugs may be used to manipulate our body's natural defense system to be more effective and help thwart diseases where T cells are too active or not active enough. T cell mechanobiology will also have practical application in T cell immunotherapy. T cell immunotherapy encompasses taking the patient's T cells out of the body, reprogramming them to attack cancer cells, and then expanding them *ex vivo* before injecting them back into the patient. Optimizing the mechanics of the *ex vivo* culture to achieve an adequate T cell expansion is essential for immunotherapy, and especially important in the case of leukemia patients who have very few T cells available in their blood.^25^

In this review, we describe some of the unique characteristics of T cells in Sec. II. Section III is presented as a tutorial with a specific focus on experimental design choices to activate T cells in engineered culture systems. We then describe the mounting research that has shown that T cells are mechanosensitive, beginning with studies employing T cells in 2D culture systems. This is followed with a description of engineering strategies to perturb and quantify T cell forces in the so-called 2.5D and 3D culture systems. Finally, we look at potential future techniques that could be used to study T cell mechanotransduction and how the findings from this emerging field may lead to an optimization of culture environments for T cell expansion and an overall greater immunotherapeutic potential for cultured T cells.

## II. INTRODUCTION TO T CELL BIOLOGY

Sections II A–II D are written as a quick introduction for those who are not familiar with T cell biology. This brief tutorial is not meant to be comprehensive, but rather focuses on topics that may be critical to consider when evaluating past T cell mechanotransduction studies and/or designing future mechanotransduction studies. In this section, we discuss the role of T cells in the body, their life cycle and a description of the processes following antigen recognition. For the interested reader, many excellent review papers providing greater depth on T cell immunology are available elsewhere.^15,26^

### A. Adaptive immunity and autoimmunity

T cells, also commonly referred to as T lymphocytes, are key players in the adaptive immune system and are responsible for triggering a host response in the presence of antigen presenting pathogens and tumor cells. Since these cells are responsible for initiating the immune cascade, it is critical that they are able to accurately distinguish between self and non-self for efficient self-defense. In the absence of this distinction, T cells may incite an immune response against the host resulting in an “autoimmune disease” wherein the body begins to attack its own cells, as is the case in diseases such as type 1 diabetes (T1D) and multiple sclerosis (MS).

### B. T cell subsets: CD4^+^ and CD8^+^

There are two major subsets of T cells: CD4^+^ and CD8^+^, which are distinguished based on the type of major histocompatability complex (MHC) that the T cell recognizes. MHC molecules are displayed on the surface of the target cell and can be categorized as either MHC class I or II. CD8^+^ T cells, also known as cytotoxic T lymphocytes (CTLs), recognize MHC class I, which is displayed on all nucleated cells in the body. Once a target cell is identified, CTLs bind to the target cell and induce apoptosis by releasing lytic granules containing the toxic proteins perforin and granzyme, which bore pores in the lipid bilayer of the target cell. Alternatively, CD4^+^ T cells recognize MHC class II, which is expressed by specialized immune cells called APCs. In general, CD4^+^ T cells are tasked with activating other cells of the immune system. Their functions involve helping B cells to produce antibodies, inducing macrophages to enhance their microbicidal activity, and recruiting other types of immune cells such as neutrophils to an inflammation site. Because of these “assistor” functions, they are also referred to as T helper cells. CD4^+^ T cells can further differentiate into subsets, with the four most prevalent being type 1 T helper cells (Th_1_), type 2 T helper cells (Th_2_), T follicular helper cells (T_fh_), and type 17 T helper cells (Th_17_). These subsets also have specialized functions. For example, Th_1_ responds to infections caused by intracellular bacteria, whereas Th_2_ responds to extracellular parasites. The discussion here will focus on Th_1_, whose response to inflammation is well documented and whose mechanotransduction pathways are the most understood. When Th_1_ cells identify a target cell expressing an antigen cognate to their TCR, they bind to it and start secreting cell-signaling proteins known as cytokines. Th_1_ cells typically express interferon-gamma (IFN-γ), interleukin 2 (IL-2), and tumor necrosis factor (TNF), which orchestrate the immune cell-mediated response.

### C. Life cycle of the T cell

The life cycle of T cells begins in the thymus where they differentiate into either the CD4^+^ or CD8^+^ subsets. Afterwards, the T cells migrate to the secondary lymphoid tissues [e.g., lymph nodes (LNs)] where they are activated after encountering their cognate antigen, expand, and finally differentiate into either an effector subset (i.e., Th_1_, Th_2_, T_fh_, Th_17_, and effector CD8^+^) or a memory cell. A T cell identifies a particular APC using the TCRs presented on its surface that identifies cognate antigen-peptides coupled to MHC (pMHC). Immediately after the TCR identifies the pMHC, the T cell binds to the APC and an activation process is triggered. The activation process leads to formation of a stable contact with the APC and initiates a cascade of events that includes TCR phosphorylation, cytoskeletal reorganization, Ca^2+^ influx, and cytokine production. After approximately 3–4 days of contact with the APC, these effector T cells leave the LNs and travel to the site of infection to further orchestrate the immune response.

### D. Immunological synapse

**FIG. 2.**
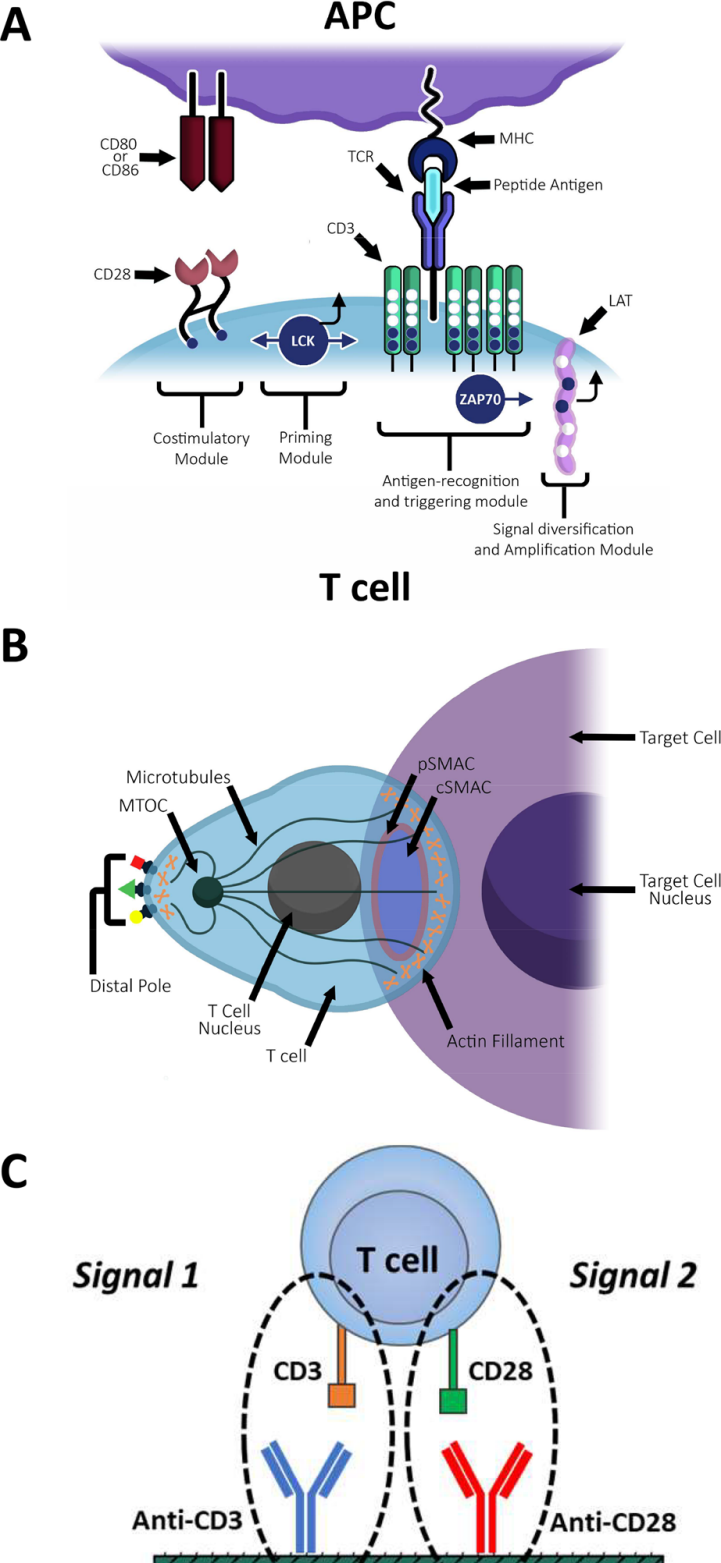
TCR downstream signaling and cytoskeletal reorganization. (a) Structure of the TCR module and early downstream signaling. Upon TCR recognition of an antigenic peptide loaded onto MHC (pMHC), phosphorylation of CD3 ITAMs (open blue circles) by the protein tyrosine kinase LCK leads to the recruitment and activation of ZAP70, which in turn phosphorylates tyrosine residues (filled blue circles) found in the cytoplasmic segment of the linker for activation of T cells (LAT), amplifying and diversifying the seminal signal. The CD28 costimulator recognizes CD80 or CD86 ligands at the surface of the APC. (b) Cytoskeletal reorganization following TCR stimulation. Following TCR stimulation, filamentous (F)-actin polymerization is induced at the IS, and the T cells microtubule-organizing center (MTOC) is polarized. A mature IS has a typical bull's-eye pattern consisting of concentric rings of membrane receptors: the inner circle, the central supramolecular activation cluster (cSMAC), and the peripheral supramolecular activation cluster (pSMAC). This pattern will occur when Th_1_ T cells contact B cells, tumor cells, and artificial APCs (aAPCs), but not when contracting dendritic cells (DCs).^29^ At the opposite pole to the IS, a less well understood protein complex called the Distal-Pole Complex (DPC) is formed. The DPC consists of the cell-surface receptor CD43 and also involves F-actin polarization to the rear side of the T cells. (c) *In vitro* T cell activation. Only two signals are needed to activate T cells artificially *in vitro*, TCR and CD28 (costimulator) stimulation. Substrates (e.g., beads, gel surfaces) can be functionalized with stimulatory antibodies for these receptors.

To understand how physiological T cell activation occurs, we first need to familiarize ourselves with the TCR module and CD28 costimulator ligands (Fig. 2). Costimulator ligation is essential for T cell activation, as TCR stimulation without costimulation will lead to cell unresponsiveness, or anergy. The TCR module is a transmembrane complex consisting of CD3 protein subunits, sometimes denoted as the TCR/CD3 complex. The intracellular component of the CD3 contains immunoreceptor tyrosine-based activation motifs (ITAMs). Once a TCR is ligated by a pMHC, the lymphocyte-specific protein tyrosine kinase (LCK) is activated and simultaneously initiates two signaling cascades. First, LCK phosphorylates the CD3 ITAMs, which create a docking site for Zap70, a protein critical for T cell activation. Zap70 is recruited to the docking site and activated to phosphorylate the cytoplasmic segment of the adaptor protein linker for activation of T cells (LAT), which in turn controls signal amplification and diversification downstream of the TCR.^27^ In the second signaling cascade, LCK phosphorylates the cytoplasmic tail of the costimulator protein CD28, a critical step for proper functionality of the CD28 surface receptor.^26,28^

Following TCR-pMHC binding, CD28 stimulation through APC receptor CD80 is the second signal required for activation. Stimulating the TCR and CD28 will direct the T cell to form an IS with the APC.^30^ In contrast, ligation of CD28 alone will lead to the induction of inhibitory signals in T cells, and TCR binding alone results in either apoptosis or a state of anergy.^31^ T cell polarization and IS maturation begins about 5–10 min after the IS has formed. This process includes T cell spreading across the APC and the formation of micrometer-scale clusters of a variety of cell receptors, including TCR and CD28. The TCR/CD3 complexes accumulate at the center of the IS to form the central supramolecular activation complex (cSMAC), while microclusters of lymphocyte function-associated antigen 1 (LFA-1) that promotes cell adhesion enclose it, creating the peripheral SMAC (pSMAC)^32^ [Fig. 2(b)]. As these receptor-microclusters migrate across the IS, their corresponding counterpart receptors on the APC surface [pMHC and intercellular adhesion molecule-1(ICAM-1)] move in a complementary manner. This surface ligand mobility on the APC can be an important experimental design parameter for studying how mechanical cues affect T cell activation.

## III. EXPERIMENTAL METHODS TO ACTIVATE T CELLS AND STUDY THEIR RESPONSE OUTCOMES OVER TIME

Sections III A and III B are written as a brief tutorial for those who are unfamiliar with *in vitro* methods of T cell activation, particularly focusing on stimulation approaches and the resulting time line of cell responses. The design of the experimental setup directly affects the T cell activation response and needs to be carefully planned depending on the research hypothesis. Thus, this section serves to present key concepts critical for understanding current research done in the field of T cell mechanobiology.

### A. Current methods to activate T cells

The mechanobiology of T cell activation is an increasing area of scientific research. A key requirement for any experiment in this field is the ability to induce T cell activation in a reproducible manner. Using natural APCs [i.e., macrophages, B cells, or dendritic cells (DCs)] in combination with their cognate antigen for T cell stimulation is the most physiologically relevant approach to emulate native conditions. However, practical challenges of this approach include the typically low yield of isolated cells using laborious, expensive processes requiring specialized equipment. Another major challenge with cell-mediated activation is the large variety of cellular subsets. Because different cell subsets can modify the kinetics and the magnitude of T cell activation responses, isolated APCs must be carefully analyzed and sorted to achieve reproducible, quantitative data.^33^ As a result, simplified approaches to activate T cells are often currently used in laboratories [Fig. 2(c)].

Depending on the activation method, T cells may proliferate at different rates, secrete different cytokine repertoires, and release these cytokines with unique temporal profiles.^34–36^ Therefore, choosing the appropriate activation method is crucial for the design of the experimental set-up. Here, we will discuss some of the current methods to stimulate naïve T cells and how they differ in terms of their physiological relevance, required stimulation time, TCR dependency, and antigen specificity.

#### 1. Phorbol myristate acetate (PMA) and ionomycin (Iono)

PMA and Iono are small organic molecules that diffuse into the cytoplasm through the cell membrane. When used together they directly activate protein kinase C (PKC) and raise the intracellular level of Ca^2+^, which triggers the calcium release required for the nuclear factor of activated T-cells (NFAT) signaling. Five hours of stimulation with these two chemicals is enough to achieve complete T cell activation and to induce sustained production of IFN-γ, IL-2, and IL-4.^35,37^ However, because this method completely bypasses TCR stimulation and signaling, this method is nonphysiological, not TCR dependent, and not antigen specific. Furthermore, this method upregulates the Fas ligand, which is involved with cell death in T cells, and thus is toxic to them over long incubation times.^34,38^ While small molecule activation with PMA is a type of purely chemical activation, all other methods of T cell activation (discussed later) include both a biochemical and a potential biomechanical component, since receptor-ligand binding interactions are involved.

#### 2. Artificial APC-mimicking interfaces

Two signals are necessary for *in vitro* T cell activation by artificial APC (aAPC): first TCR engagement and then binding of the costimulatory receptor [Fig. 2(c)]. A variety of strategies have been developed to attempt synapse formation in 2D and 3D utilizing aAPCsurfaces, including the use of antibodies or pMHC tetramers on 2D or 3D surfaces.^39^ For 3D strategies, the surfaces can be either non-living or cell-based.^40,41^ Below is a brief compilation of the key features, advantages and limitation for each of these aAPC systems, which have been presented and discussed in greater detail elsewhere.^41,42^

The 2D and 3D surfaces of aAPCs can be designed to precisely fine-tune activation signal strength by modulating several parameters: the TCR-ligand and costimulatory-ligand surface density, ligand affinity, and the cytokine milieu.^43^ A common strategy utilizes monoclonal antibodies (mAbs) and pMHC-tetramers as stimulating ligands for the TCR signaling pathway.^44^ Specifically, anti-CD3 and anti-CD28 mAbs are used to artificially mimic TCR-dependent activation by artificially aggregating the receptors in the membrane and binding the CD3ε signaling subunits. They deliver a much stronger signal than the physiological cognate ligand pMHC and lead to a robust polyclonal activation response.^40^ One reason for this particularly strong activation response can be attributed to anti-CD28 mAbs lack of ability to bind to the inhibitory receptor CTLA-4 that negatively regulates T cell activation. Comparatively, the pMHC-tetramer is antigenic peptide specific, thus more physiologically relevant.

Both of these methods induce cytokine burst-release profiles^35^ and can be used for long incubation periods. Interestingly, anti-CD3 and anti-CD28 mAbs conjugated to the surface of 3D beads induce greater levels of cytokine secretion than the corresponding 2D plate-bound aAPC.^45^ This result was hypothesized to be due to the larger surface area that the geometry of the bead offers compared to the 2D plate. Geometry is a critical component in designing the shape of the aAPC to improve T cell activation. The increased aspect ratio of ellipsoidal microparticles conjugated with anti-CD28 mAbs and pMHC with particle volume and antigen content held constant also resulted in enhanced T cell activation over the comparative spherical aAPC. The long axis of the ellipsoidal aAPC led to increased interaction with CD8^+^ T cells.^46^ A main limitation of most 2D and 3D aAPCs is that they are static, and thus do not mimic the changes in geometry, ligand configuration, and surface stiffness that naturally occur in APCs during IS formation.^11^ Importantly, the surface rigidity of the aAPC has been shown to mediate the amount of force that T cells can exert.^47^ This effectively limits the dynamic mechanical feedback experienced by the T cells, which is likely a critical component of this mechanotransductive process.

Cell-based aAPCs are engineered cell lines transfected using a retrovirus or lentivirus to express the pMHC and the costimulator receptor. Notable examples include the K562 human erythromyeloid line^48^ and the murine NIH/3T3fibroblast line.^49^ Unlike most static, rigid acellular-aAPCs, these cell-based aAPCs can have dynamic responses and mechanical properties that are more similar to the native APC. However, because of their inherent complexity, cell-based aAPCs have not yet been used in the context of mechanotransduction studies and are reviewed elsewhere.^40,41,50^

### B. Activation response outcomes

**FIG. 3.**
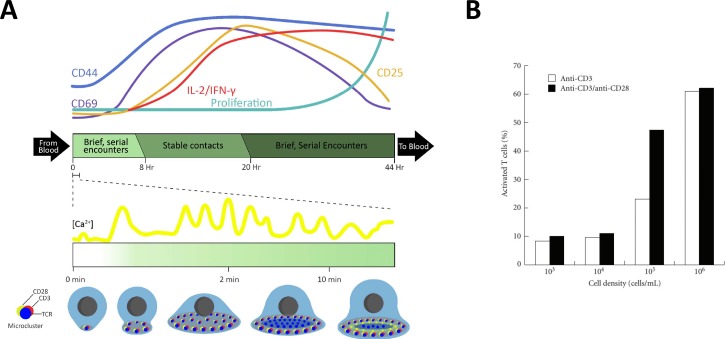
T cell activation. (a) Outcomes and time course of T cell activation. Cytoplasmic calcium concentration will increase within seconds after IS formation; T cell polarization and IS maturation begins about 5–10 min after the IS has formed. This process includes T cell spreading across the APC and the formation of micrometer-scale clusters of a variety of cell receptors, including TCR, CD3, and CD28. Within about 2–4 h, cell surface activation-markers, i.e., CD69, CD44, and CD25 are upregulated, followed by increased secretion of cytokines, such as IL-2 and INF-γ. T cells start proliferating 24–48 h after TCR stimulation. (b) Cultured density of T cells significantly affects T cell activation. T cells were activated in the presence of IL-2 either with anti-CD3 alone or together with anti-CD28 and quantified after six days in culture to determine the percentage of T cells exhibiting an activated “blast” morphology. Reproduced with permission from Ma *et al.*, J. Biomed. Biotechnol. **2010**, 386545. Copyright 2010 Hindawi Publishing Corporation.^55^

The activation response manifests itself in two phases: early and late activation, the timeline of which is described graphically in Fig. 3(a). Sections III B 1 and III B 2 will discuss features and time-points characteristic of the activation response with a focus on those that can be observed and quantified in engineered systems.

#### 1. Early activation

##### a. Migration

Antigen-specific T cells are very rare, and dendritic cells (DCs) maintain a low frequency (approximately 1% of the LN cells), as a result T cell motility plays a key role in locating antigens from APCs and targeting cells. T cells can modify their motility patterns based on environmental cues and current level of activation.^51^ For example, increasing T cell motility increases the probability of encountering a target cell and triggering TCR, whereas decreasing motility allows for the formation of a more stable IS with a given target call. As soon as a T cell identifies a cognate APC, it decelerates from >10 *μ*m/min to <2 *μ*m/min and fully arrests upon forming a stable IS.^52^ Stimulation of naïve T cells in the LN is organized into distinct phases: (1) brief interactions with the APC (<7 min), followed by (2) a stable contact phase with prolonged interaction and arrest (∼30 min), and (3) a final phase of serial, brief interactions in which motility is restored and the T cells proceed to the inflammation site (Fig. 1).^53^ When finally reaching the target tissue, the T cell motion is described as amoeboid, reaching speeds up to 20 *μ*m/min; this remarkably fast motility is thought to be enabled by weak adhesion to the matrix.^54^

##### b. Cytoskeletal changes

Many of the processes essential to initiate and sustain T cell activation response are cytoskeleton-dependent.^30^ These include integrin-mediated adhesion, IS formation and maturation, cellular polarization, Ca^2+^ flux, receptor signaling, and downstream changes in gene expression. The cytoskeleton regulates T cell activation by serving as a platform for the recruitment of molecules that regulate adhesion and signal transduction, such as Zap70 and LAT.^56^ Cytoskeletal reorganization follows TCR stimulation and includes actin polymerization and accumulation in the IS, reorientation of the microtubule-organizing center (MTOC) towards the region of cell-cell interaction,^57^ and the formation of an actin-rich structure known as the distal-pole complex on the opposing side of the cell^58^ [Fig. 2(b)]. During cell activation, the actin filaments can be polymerized or depolymerized in a dynamic manner as a means of regulating the mechanical forces needed to sustain activation and motility.^59^ Disruption of the cytoskeleton impacts T cell activation,^13,56,60^ and inhibition of actin polymerization after the IS has formed diminishes the activation response.^61,62^ While this is only a brief summary of cytoskeletal rearrangements during T cell activation, several thorough reviews of this highly complex interaction can be found elsewhere.^62–64^

##### c. Ca^2+^ flux

Calcium signaling is an early event following IS formation. The Ca^2+^ concentration within the cytoplasm dramatically increases within seconds following initial APC-T cell contact [Fig. 3(a)].^65^ In effect, the influx of Ca^2+^ serves as a “stop signal” and initiates the steps necessary to reduce T cell motility. This Ca^2+^ increase occurs in parallel with TCR microstructure formation and is a necessary step in rapid cytoskeletal reorganization^66^ to accommodate the formation of a stable IS.

##### d. Activation markers and gene expression

CD44 is a surface glycoprotein that is a known receptor for hyaluronic acid. However, CD44 can also bind to other ligands, such as collagens and matrix metalloproteinases (MMPs), and is involved in cell-cell interactions, cell adhesion, and migration. CD69 is a surface glycoprotein that functions as an immunoregulatory molecule during immune response.^67^ Both of these glyocoproteins are upregulated during the brief serial encounters with APCs in phase one^68^ and are rapidly expressed after TCR stimulation, peaking after approximately 24 h [Fig. 3(a)]. The CD69 activation marker has been correlated with the strength of the activation response, and its upregulation is a highly sensitive measure of antigen recognition. However, while CD44 is continually expressed, CD69 expression rapidly declines following the end of TCR stimulation.^69^ While these glycoproteins are useful markers to quantify the T cell activation response, one needs to be careful as they can be upregulated even when stimulation does not reach the minimum threshold for complete T cell activation.

NFAT and nuclear factor kappa-light-chain-enhancer of activated B cells (NF-kB) are transcription factors that are expressed within half an hour following TCR stimulation. The expression of these transcription factors is independent of the formation of a stable T cell-APC conjugate and are expressed during brief serial APC encounters in phase one.^70^ The induction of NF-kB is crucial for substantial production of the cytokine interferon-γ (IFN-γ).^71^

#### 2. Late activation

##### a. Cytokine expression: Interferon-γ (IFN-γ) and interleukin-2 (IL-2)

Cytokine secretion is necessary for regulating the body's inflammatory response. Two main cytokines are secreted when Th1 cells and CD8^+^ T cells are activated: IFN-γ and IL-2, both of which are expressed intracellularly and then secreted out of the cell.^72^ Intracellular expression is rapid, and can be detected anywhere between 30 min to 2 h following APC-T cell conjugation. However, we will focus on the secreted form, which can be detected only at later time points (>3–8 h) [Fig. 3(a)]. IFN-γ, named for its ability to interfere with viral infection, is a potent activator of macrophages and plays a central role in inflammation and autoimmune disease and is produced during the brief sequential encounters with APCs, about 3–6 h after initial contact. IL-2 is critical for the stimulation of T cell proliferation, differentiation, and survival. Thus, it is also essential for *ex vivo* T cell expansion.^73^ In comparison to IFN-γ, IL-2 production requires a prolonged stable contact^74^ and is secreted 2–4 h after a stable IS has formed.^75^

##### b. Activation marker—CD25

CD25, also known as the IL-2 receptor, is an activation marker expressed on the T cell surface. CD25 expression is already induced during the brief sequential encounters that occur in phase one. Its expression can be detected 24 h after TCR stimulation and peaks at 48 h, but it is rapidly lost after 72 h [Fig. 3(a)].^76^

##### c. T cell proliferation

T cell proliferation starts 24–48 h after TCR stimulation, and in culture systems, the T cell proliferation is significantly affected by the density of T cells and APCs [Fig. 3(b)]. Low cell density leads to infrequent cell-cell contact, which results in a deficiency of CD3 crosslinking. As a result, naïve T cells die quickly by apoptosis when seeded in low cell densities (<1 × 10^4^ cells/ml), but can survive for extended periods when cultured in 2D at high density (>1 × 10^6^ cells/ml), which promotes higher levels of T cell activation and proliferation.^55,77^ In addition to T cell density, the duration of stimulation is a major factor in determining the fate of naïve T cells.^78^ Naïve T cells require approximately 20 h of sustained contact to be fully activated and to start proliferating. This time duration includes the first sequential encounter phase, which is inversely correlated with APC density and the number of pMHC per APC,^77^ and the stable contact phase. Chronic stimulation *in vivo* or overstimulation *in vitro*, usually more than 3 days, carries the risk of inducing cell death.^78,79^

## IV. CURRENT TOOLS AND FUTURE OPPORTUNITIES TO EXPLORE T CELL MECHANOBIOLOGY

**FIG. 4.**
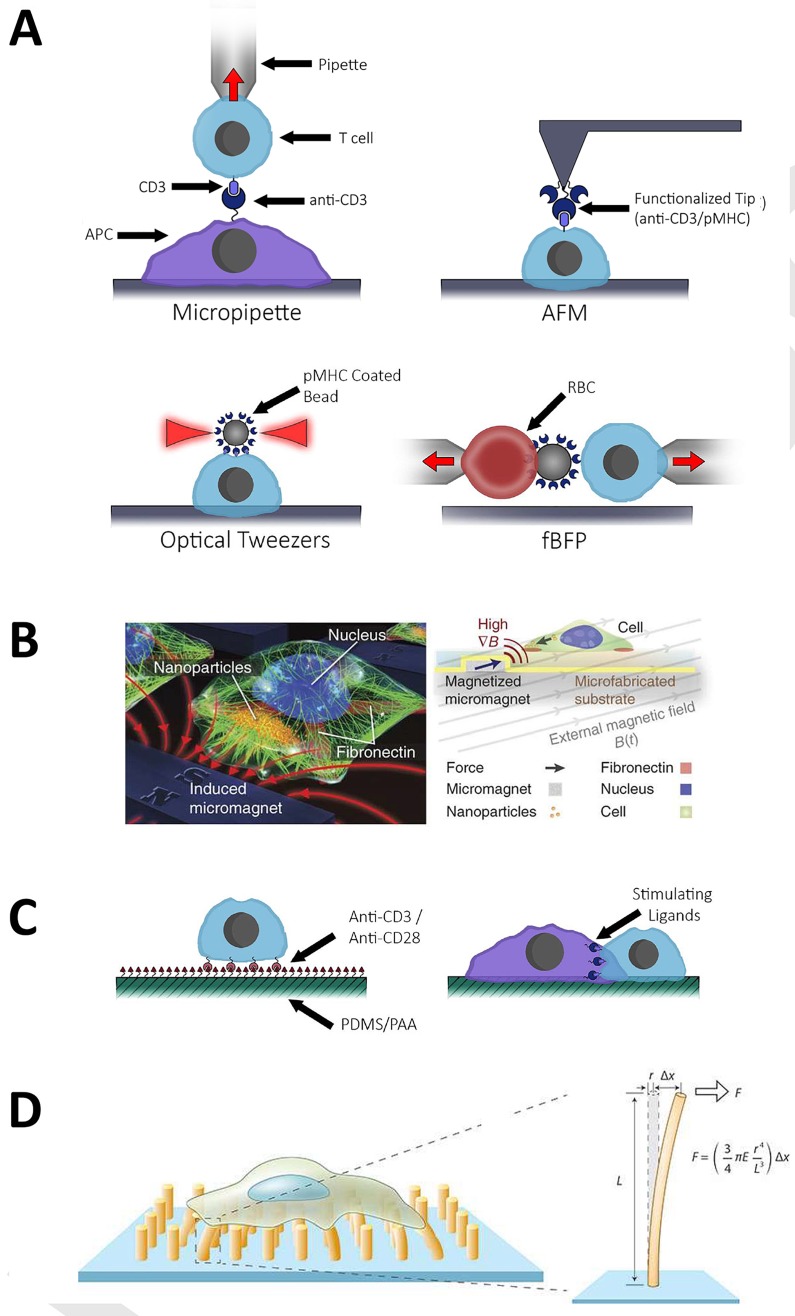
Potential bioengineering strategies to study T cell mechanotransduction. (a) Methods using localized forces to probe single cells. A *micropipette* brings a T cell into contact with an antigen presenting cell (APC); the micropipette can pull the T cell and induce normal or shear forces. The tip of an *atomic force microscope* (AFM) can be functionalized with either pMHC or anti-CD3 to deliver a stimulatory signal to an adherent T cell while applying force-loads in specific patterns and time-durations. *Optical tweezers* can apply a directional force on an adherent T cell using coated beads, commonly functionalized with antigenic peptide loaded onto MHC (pMHC). The *fluorescence biomembrane force probe* (fBFP) strategy includes a functionalized bead that is attached to the apex of a micropipette-aspirated red blood cell (RBC) and a T cell that is also micropipette-aspirated. The latter brings the T cell and the bead into contact while precisely controlling the distance between them and the external forced applied. (b) Magnetic force-based platform. Magnetic fields are applied to cells dosed with magnetic nanoparticles. This technique enables on-demand exertion of localized force over a population of cells. Reproduced with permission from Tseng *et al.*, Nat. Methods **9**(11), 1113 (2012). Copyright 2012 Nature Publishing Group.^92^ (c) Methods using synthetic polymer substrates with variable stiffness. T cells are stimulated through antibodies (i.e., anti-CD3, anti-CD28) that are conjugated to the substrate surface (left side) or through APCs that are seeded together with the T cells on the substrate surface (right side). (d) Micropost array platform. Polymeric microposts bend in response to cell-applied traction forces (F), which can be estimated based on the micropillar height (L) and radius (r), pillar mechanical properties (Young's modulus E), and the amount of bending or deflection of the pillar (ΔX). Image provided courtesy of the Mechanobiology Institute, National University of Singapore.

Mechanosensing is the act of converting mechanical cues from the external environment and converting them into biochemical signals that affect cell fate and function. As a T cell migrates through its microenvironment, it undergoes both cell-cell and cell-matrix interactions that provide these mechanical cues and influence cell fate. However, investigating how these cues influence cell fate is challenging due to the difficulty of decoupling these signals or controllably studying them together to elucidate meaningful, reproducible data. For example, a few critical factors that may cross-interact to influence T cell fate include matrix stiffness, the timing of cell-APC contact, and the matrix structure, such as topography and porosity. These individual cues often drive T cell fate and function but are hard to controllably tune within the laboratory either individually or together to gain meaningful conclusions about the causal relationships of the mechanotransduction process. To overcome these limitations, researchers have tried to study T cell mechanobiology in artificial cellular environments where they can control specific biophysical and biochemical parameters independently. In this section, we will discuss some of the tools and technologies (Fig. 4) that have been developed to study this elusive system and how they offer varying degrees of control over some relevant physiological parameters such as stiffness, type of activator ligands, the binding moieties, and the seeded cell densities.

### A. Emerging methods to explore T cell mechanobiology using localized forces

Two major observations led to exploration of the TCR as a possible mechanosensor. First, it was observed that soluble monovalent pMHCs bind to the TCR but fail to induce TCR signaling,^80^ whereas, soluble pMHC tetramers showed some degree of success.^81^ Second, a T cell and an APC perform a “dance” when they interact, during which the T cell pushes and pulls the APC's plasma membrane, thus potentially exposing the TCR to significant forces.^82^ Upon observing this forceful interaction, several researchers began speculating that mechanical cues may trigger TCR signaling by driving conformational changes in the TCR/CD3 complex.^17,83^ This led to a series of studies that utilized methods to exert localized forces on T cells to probe the nature of this interaction.

Kim *et al.*^84^ used optical tweezers (method reviewed elsewhere^85^) to manipulate pMHC-coated beads to apply a directional force of 50 pN on the T cell membrane [Fig. 4(a)]. Tangential (shear) and perpendicular (normal) forces were applied, but only the tangential force initiated Ca^2+^ flux, suggesting that the TCR/CD3 complex is an anisotropic force sensor. Later papers would challenge this result,^17^ but a proposed possible explanation is that tangential movement is better at uncovering shielded TCRs, which are covered by a thick layer of large glycoproteins, than vertical movement.^86^ Uncovering the shielded TCRs would increase the possibility of a pMHC-coated bead binding to the TCR, and therefore yield the observed Ca^2+^ flux. A follow up study by the same lab used optical tweezers to investigate how applied force-loads and force-directionality affect the chemical threshold of TCR stimulation.^87^ The pMHC surface concentration of the coated-bead was carefully manipulated and controlled. The results indicated that in the absence of applied force, the pMHC surface concentration required for activation is orders of magnitude higher than the physiologically relevant concentration. In contrast, an applied force of 10–20 pN in the shear direction can stimulate TCR activation with as little as two pMHC present.

In complementary work, Li *et al.* developed an experimental setup that attempted to increase the probability of TCR-ligand binding and thereby eliminate the confounding effects of mechanical force on TCR unshielding.^17^ In their work, the authors used a micropipette (technique reviewed elsewhere^88^) to pull and induce shear forces on T cells that were brought in contact with fibroblast-based aAPCs, which expressed a membrane-tethered, anti-CD28, co-stimulatory ligand and defined anti-CD3-ligands [Fig. 4(a)]. There were two types of TCR-ligands, a short one and an elongated one. In principle, the probability of encountering the TCR is higher for the elongated TCR-ligand because of its extended reach. However, results indicated that in the absence of external forces, the elongated TCR-ligand resulted in poor activation, whereas the short TCR-ligand led to robust Ca^2+^ flux and T cell proliferation. In contrast, in the presence of external forces, the elongated TCR-ligand successfully resulted in increased Ca^2+^ flux for both tension forces and shear forces. Furthermore, these experiments provided an indication that the process may be Src kinase-dependent. In summary, unlike the earlier report, this manuscript concluded that the TCR may not be anisotropic.

Liu *et al.* followed up on this work by developing a newly modified fluorescence biomembrane force probe (fBFP) technology to quantitatively investigate the strength of the TCR-pMHC bond in the presence of external forces.^89^ Specifically, they focused on the *in situ* kinetics of the TCR-pMHC interaction to determine whether force plays a role in antigen recognition and discrimination, which is critical for adaptive immunity. To achieve this, they expanded on BFP, a previously developed technology that measures receptor-ligand binding kinetics for a single-molecule,^90^ and modified it to also simultaneously monitor the Ca^2+^ response resulting from this binding interaction. The experimental setup [Fig. 4(a)] includes a pMHC-biotinylated glass bead that is manually attached to the apex of a micropipette-aspirated red blood cell (RBC). Another micropipette aspirates a primary, naïve T cell to control contact between the T cell and the functionalized bead. While this micropipette-based approach enables nanometer precision in cell-ligand positioning, it requires a laborious and sensitive process of manual production and assembly.^91^

The RBC is used as a sensitive force transducer, whose spring constant is calculated based on the aspiration pressure and a series of radii including the probe micropipette, (*R*_p_), RBC (*R*_0_), and circular contact area between the RBC and probe bead (*R*_c_). While the T cell and bead are brought into contact, Ca^2+^ imaging is used to detect early T cell activation, thus enabling them to establish the connection between the external force, bond characteristics, and early activation in T cells. The authors discovered that the pMHC–TCR bond is a “catch bond,” (i.e., increased force prolongs the bond's lifetime). In contrast, the antagonist ligand-TCR is a “slip bond,” (i.e., increased force shortens the bond's lifetime) which may help elucidate the mechanism of self-versus non-self-discrimination. In addition, the timing and magnitude of applied force was also found to be critical for activation, and the latter observation has been confirmed by other studies.^93^ The fBFP technique was also used by Husson *et al.*, who investigated how force-exertion of the T cell changes in response to different stiffness of the aAPC.^47^ They used an antibody-coated bead aAPC and indirectly modeled different stiffness by changing the aspiration pressure of the RBC, which effectively varies the spring constant of the force probe between 50 and 1000 pN/mm. The study demonstrated that the force exerted by the T cell during the pulling phase increases with higher force-probe stiffness, which may imply that a T cell can adjust its force response based on APC mechanics. Later, Pryshchep *et al.* used the fBFP technique to apply cyclic force-load patterns with varied intermission time and constant contact time on the TCR of a naïve T cell using a pMHC biotinylated RBC.^94^ The study found that a 5-s intermission time was sufficient to induce Ca^2+^ flux, while a 10-s intermission time impaired this ability. For all of these various fBFP studies, the experiments were performed inside a glass chamber filled with the medium. In the future, it may be interesting to perform fBFP experiments in the presence of other mechanical environments such as hydrogels or other mechanically modular environments to better recapitulate the natural system.

Another approach to quantify the forces involved in early T cell activation is the use of a functionalized AFM tip [Fig. 4(a)]. Hu *et al.* used this method to deliver an antigenic signal (pMHC) or anti-CD3 signal to primary and effector T cells to evaluate the role of force in mediating actin cytoskeleton rearrangement.^59^ T cell activation is known to be prevented in T cells with altered actin cytoskeleton dynamics.^63^ AFM was used to measure the forces generated by the T cell as well as to apply force-loads in specific patterns including cyclic, continuous, and spaced, while Ca^2+^ flux was monitored. As expected, treatment of the T cells with latrunculin A (LatA), which prevents polymerization of actin filaments, inhibited the ability of T cells to exert pushing and pulling forces and prevented Ca^2+^ flux upon TCR-engagement. However, upon application of a cyclic force-load, LatA-treated T cells displayed partial recovery of TCR-induced Ca^2+^ flux. Application of a cyclic force-load alone, without TCR engagement, was not successful in inducing Ca^2+^ flux, supporting the idea that mechanical forces were transmitted to the T cell through the TCR. Interestingly, continuous and spaced force-loads did not yield Ca^2+^-flux recovery in LatA-treated cells upon TCR-engagement. These data suggest that the dynamics of actin cytoskeleton rearrangement plays a key role in T cell-force generation and subsequent Ca^2+^ flux induction. The fact that only a cyclic force-load successfully initiated Ca^2+^ flux may imply that the interval time between sequential encounters needs to last less than 10 s to allow for signal integration. This AFM experimental design uses commercially available equipment and thus enables a fairly high throughput analysis of multiple, single-cell events for robust statistical analysis compared to fBFP. However, this evaluation of many different single-cell events also raises a new technical concern, that one must standardize the level of T cell activation for each individual, single-cell experiment. This includes quantification and standardization both of the level of pre-activation and stimulation. Here, a population of T cells were pre-stimulated in bulk for 2 days and then allowed to rest for 4 days prior to AFM evaluation. Thus, it is possible that not all cells had similar levels of pre-activation. Furthermore, each single-cell experiment used a freshly prepared AFM tip without quantification of ligand conjugation. As the ligand concentration can influence the magnitude of T cell activation, it is also possible that different cells were presented with different levels of activation. Nevertheless, this study demonstrates that controlled AFM-delivered forces can transform an incapacitated T cell to be operable by replacing the role of the actin cytoskeleton in force-actuation.

This section discussed methods that can be used to mechanically probe a single cell, including optical tweezers, micropipette aspiration, fBFP, and AFM. These methods can assist to examine short and early signaling events after TCR stimulation but are limited in the exploration of late activation signaling, such as cytokine secretion and proliferation, which occurs during long incubation periods and are influenced by a threshold density of T cells. Furthermore, it may be challenging to keep a single T cell viable for long time periods while being probed with these techniques. That being said, there might be a way to circumvent this challenge by adding different combinations of cytokines to the cell medium that will facilitate delivery of survival cues to the T cell.

One potential future opportunity to overcome some of the limitations of the mechanical probing strategies used thus far in T cell research is techniques based on magnetic tweezers. Recently, Tseng *et al.* developed a novel strategy to control localized, on-demand, time-varying exertion of force over a population of cells. In this method, they used individually patterned magnetic nanoparticle-dosed cells and applied magnetic fields to achieve localized and spatially resolved forces, which can be maintained for extended periods of time on the cell membrane [Fig. 4(b)].^92^

Finally, most of these experimental setups have used an aAPC in the form of a functionalized glass bead or a silicon AFM probe, both of which have stiffness in the GPa range, which is non-physiological. As an alternative approach, researchers have begun to use 2D polymeric substrates, which are the focus of Sec. IV B, to evaluate T cell responses to physiological stiffness changes over long activation time periods.

### B. Emerging methods to explore T cell mechanobiology using 2D substrates

Judokusumo *et al.* formed 2D, polyacrylamide (PAA) gel substrates with different material stiffness (2–200 kPa) by modulating the crosslinker density.^19^ The substrates were coated with anti-CD3 and anti-CD28 antibodies using streptavidin-biotin conjugation, and naïve murine CD4^+^ T cell activation was monitored by quantifying IL-2 secretion [Fig. 4(c)]. Interestingly, there was no difference in IL-2 secretion for substrates with moduli lower than 10 kPa, possibly implying that a minimal substrate rigidity is required for effective mechanotransduction. To study whether this response is actin cytoskeleton-mediated, they treated the cells with a myosin inhibitor, blebbistatin. Blebbistatin-treated samples showed reduced IL-2 secretion on samples with Young's moduli higher than 10 kPa. They further suggested that this mechanotransduction effect may be mediated by phosphorylation of the Zap70 protein and the Src family kinase proteins (SFK), which are known to be downstream from TCR stimulation.

Motivated by immunotherapy applications, O'Connor *et al.* investigated the effect of substrate rigidity on the expansion of human CD4^+^ and CD8^+^ T cells using poly(dimethylsiloxane) (PDMS) [Fig. 4(c)].^18^ They produced substrates with Young's moduli ranging from ∼100 kPa to above 2 MPa by manipulating the crosslink density, and they stimulated the cells through the adsorption of anti-CD3 and anti-CD28 ligands. Their work showed that IL-2 and IFN-γ production were significantly higher on more compliant gels (∼100 kPa). Additionally, they evaluated proliferation over a ∼15-day incubation period and found that the log phase period of cell doubling extended to 15 days on the more compliant gels, whereas on the stiffer gels this phase continued only for 10 days. These data are difficult to compare with the findings of Judokusumo *et al.*, since the modulus values for the intermediate stiffness PDMS substrates were not characterized and the range of stiffness only partially overlap between the two studies. In addition, the two reports use different polymer chemistries, which may result in different nano- and/or micro-structure of the polymer substrates and may have different levels of background cell toxicity.^95,96^ In addition, O'Connor *et al.* coated their substrates through passive adsorption, which may not be stable over long incubation times. While their data demonstrated stable binding of the stimulatory ligands for the first 2 days, further validation is required to support longer experimental times. Nonetheless, this paper highlights the importance of mechanical properties of the microenvironment as a regulator of T cell function.

The stiffness range explored in the previous papers was markedly higher than the lymphoid native tissue and APC stiffness, which typically are lower than 1.5 kPa.^11^ Hui *et al.* explored the stiffness range of 1–5 kPa using PDMS substrates treated with poly-d-lysine (PDL) to promote cell adhesion and covalent attachment of anti-CD3 ligands.^20^ They quantified the activation response of an immortalized cell line, Jurkat T cells, through immunoblotting, specifically looking at tyrosine phosphorylation. The results suggested that increased substrate stiffness leads to an increase in transient tyrosine phosphorylation, which is known to contribute to early activation events. A limitation of this study is that it lacked presentation of a costimulatory ligand, such as anti-CD28. Regardless, this paper indicates that contrary to what had been previously reported, the mechanics of compliant substrates may also affect T cell mechanotransduction.

Most recently, Saitakis *et al.* investigated the physiological range of stiffness (0.5–100 kPa) using PAA gel substrates.^97^ The substrates were coated with anti-CD3, anti-CD28, and intercellular adhesion molecule-1/FC-chimeric molecules (ICAM-1) using the streptavidin-biotin conjugation method employed previously.^19^ The study used pre-activated human CD4^+^ T cells (day 6) that were seeded on gel substrates with variable stiffness. The results indicated that T cells were more activated on stiffer substrates than on softer substrates and that some effector functions were sensitive to the whole range of stiffness examined (e.g., migration, gene expression, cytokine expression, and secretion), whereas other functions (e.g., cell cycle progression and metabolism) were only affected at later (72 h), but not early (24 h), time points. Interestingly, stiffness-induced changes in gene expression were only observed when anti-CD3 stimulatory ligands were present, which suggests that the TCR/CD3 complex may be the main mechanosensing module. In addition, cytokine gene expression and secretion were enhanced with increasing substrate stiffness with and without ICAM-1 present. This study was the first to evaluate the role of matrix stiffness within the context of an integrin ligand, ICAM-1. This is important as other studies have reported that integrins mediate the mechanotransductive process in other cell types.^98^

While most systems have investigated the effects of substrate stiffness by using ligand-functionalized synthetic materials, Basu *et al.* employed natural materials to study these interactions under more physiologically relevant conditions. To achieve this, they made two modifications to the PAA system from before: an adsorbed fibronectin coating was put on the PAA surface (stiffness range of 12 kPa and 50 kPa) and antigenic peptide ovalbumin (OVA)-loaded APCs were used to physiologically stimulate CD8^+^ T cells.^9^ The use of true APCs on top of substrates with different stiffness enabled them to decouple the matrix mechanical cues from the cell-cell interactions involved in IS formation [Fig. 4(c)]. Results indicate that CD8^+^-mediated killing, specifically the ability of T cells to bore through the membrane of the APCs, is enhanced on stiffer substrates. Therefore, this paper was the first to link T cell mechanotransduction to a physiologically functional outcome of T cell activation. One limitation of the experimental protocol is that the fibronectin was coated onto the PAA using physical adsorption rather than chemical conjugation. The cells pushing and pulling against the surface may cause the fibronectin to detach from the substrate, thus remodeling the fibronectin protein layer and causing the microenvironment's biochemical cues to vary across substrates. In addition, the uniformity and concentration of the fibronectin coating was not quantified, and this may be altered by the PAA crosslink density.^96^ T cells express receptors for fibronectin (e.g., VLA-4 and VLA-5) that are known to assist in TCR activation, but their signaling pathways and functionality is not fully understood.^99^ It is an open question whether these receptors contribute to mechanotransductive effects that mediate CD8^+^ T cell killing.

In this same manuscript, the authors also used a micropillar method to spatially resolve the forces exerted by cells on the surfaces to which they are adhered. Micropillars of defined geometry and fabricated from elastic materials (e.g., PDMS) have a known spring constant.^100^ Observing the micropillars' deflection during interaction with cells provides a means of measuring the cell traction forces. Basu *et al.* used micropillars to verify that T cells exert mechanical forces onto doomed target cells, resulting in more efficient killing.^9^ In a spatiotemporally coordinated attack, CD8^+^ T cells exert force onto a targeted cell's membrane along with a significant release of perforin. This combination of mechanical force with perforin release aids in pore formation in the targeted cell by essentially stretching out the cell membrane and thus priming it for pore formation. Activated CD8^+^ T cells exerted more force than naïve T cells and in an asymmetric pattern, suggesting a spatially targeted interaction with a target cell. These newly formed pores allow a large influx of cytotoxic proteins into the cell, thus resulting in more rapid cell death. Locations of the highest force exertion were coupled with a local increase in cytolytic protein secretion. This study showed that T cells leverage mechanical forces to aid in cytotoxic activity against target cells.

Bashour *et al.* used micropillars functionalized with anti-CD3 and anti-CD28 to demonstrate that T cells exhibit non-integrin-based mechanosensing through these activating and co-stimulating signals.^101^ The interactions between a T cell and the pillars were classified into four distinct phases: (1) contact, (2) spreading, (3) transient, and (4) contractile phases. The greatest traction forces were observed during the transient and contractile phases, with forces reaching approximately 100 pN on pillars presenting both anti-CD3 and anti-CD28. Upon stimulation with pillars displaying only anti-CD3, T cells generated about half the traction force as observed in the anti-CD3/anti-CD28 combined condition. Cells seeded on anti-CD3-only pillars with anti-CD28 present in the culture medium exerted approximately the same traction forces as the anti-CD3/anti-CD28 combined condition, suggesting that TCR-CD3 interaction is where force generation occurs, while CD28 increases the force through biochemical signaling. T cells did not significantly adhere to anti-CD28-only decorated pillars. This study concluded that T cells exert traction forces through TCR-CD3 interactions with associated signaling in the Src kinase family.

Both Basu *et al.* and Bashour *et al.* used micropillars with a single defined rigidity and geometry. In the future, researchers can employ the micropost array platform developed by Fu *et al.*, in which PDMS pillar rigidity is altered through changing their composition or height [Fig. 4(d)].^100^ This controlled system can be adopted to investigate T cell force generation in response to substrates of different rigidity. That said, studying T cell traction forces using the micropillar approach may introduce other complexities, since T cell force-generation may be limited by the pillar spacing. Previous work (discussed in more detail below) has shown that T cells are exquisitely sensitive to patterns of stimulating ligands; thus, it is unclear if forces induced by micropillars (which inherently present stimulating ligands as an arrayed pattern) would be similar to forces induced by homogeneous, 2D substrates.^102^ One possible approach to overcome this challenge in the future is traction force microscopy (method reviewed elsewhere^103,104^). This approach measures the forces generated by adherent cells on flat, elastomeric substrates. By imaging the displacement of fluorescent beads embedded near the top of the substrate, one can estimate the traction forces exerted on the substrate. A potential additional advantage of traction force microscopy is that it can be used to resolve forces applied both in the horizontal and vertical directions.^104,105^

When preparing functionalized substrates for mechanotransduction studies, one important variable that has yet to be quantitatively evaluated is the spatial organization of the activating ligands. In complementary work, spatial organization of signaling factors has been shown to have important consequences for T cell activation.^106^ For example, Shen *et al.* explored how spatial distribution of anti-CD28 and anti-CD3 cues influenced T cell activation on rigid substrates using microcontact printing.^107^ Cell behavior on microscale, circular patterns containing anti-CD3 co-localized with anti-CD28 was compared to that on circular patterns of segregated “islands” of anti-CD3 interspersed with “islands” of anti-CD28. Murine naïve T cells (CD4^+^) were observed to have greater IL-2 secretion on segregated patterns compared to co-localized patterns, which was correlated with NF-kB translocation and PKB/Akt signaling.

In another micropatterning study, Jung *et al.* demonstrated that the asymmetric division of activated T cells could be controlled by the microscale presentation of anti-CD3 and anti-CD28 with an ICAM-1 background on PDMS substrates.^108^ Asymmetric division of activated T cells is believed to be a mechanism by which a single T cell can give rise to a diversity of effector or memory T cells. In this work, the size (4 and 10 *μ*m) and distance (15 and 20 *μ*m) between activation sites of anti-CD3 and anti-CD28 were varied. A larger distance between activation sites led to higher instances of asymmetric division of CD4+ murine T cells upon activation. Furthermore, they used time-lapse microscopy to image the adhesion or migration of the two resulting daughter cells. They observed that when one daughter cell remained anchored to the pattern, the TCR remained polarized during cytokinesis and asymmetric division occurred. This work highlights the influence that microscale presentation of antigens to T cells has on the subsequent T cell expansion behavior.

In addition to microscale patterning, nanoscale patterning of stimulatory molecules has also been explored. The Spatz group utilized block-copolymer micelle nanolithography to create nanopatterns of immobilized anti-CD3.^109,110^ Briefly, in Matic *et al.*, gold nanoparticles (AuNPs) were ordered on glass substrates in a nanoarray, passivated with a polyethylene glycol (PEG) layer, and then conjugated with anti-CD3.^102b^ The steric constraints of the PEG passivation resulted in a near 1:1 ratio of antibodies per AuNP, thus providing exquisite control over nanoscale patterning. They found that anti-CD3 alone could induce CD69 expression on T cells when the interparticle distance was less than 100 nm. In general, higher activation was achieved with higher density arrays, plateauing at 316 AuNPs/*μ*m^2^. Although the anti-CD3 nanoarray did not induce measurable IL-2 production, addition of soluble anti-CD28 as a costimulator did efficiently lead to increased IL-2 production.

In another report, Spatz and coworkers further explored the response of T cells to the density and specific nanopatterning of pMHC.^109^ Deeg *et al.* determined that about 100 pMHC molecules per *μ*m^2^ was the threshold for eliciting cell adhesion and IL-2 production, which corresponds to a ligand spacing of about 115 nm. For pMHC spacing above 150 nm, a significant number of cells did not adhere. They then evaluated surfaces that included both nano-patterning and micro-patterning. When comparing substrates with extended, continuous nanopatterning to substrates with micro-nanopatterning (i.e., micrometer-scale regions with dense nanometer-scale patterning), the micro-nanopatterned substrates showed less adhesion and activation than the extended nanopatterns even while the nanoscale particle density was held constant. This finding suggests that the global ligand density is of primary importance in T cell adhesion and activation over the nanoscale ligand presentation. In the future, an interesting follow-up to these studies would be to micro- and nano-pattern flexible substrates to evaluate how rigidity and stimulatory ligand patterning co-influence T cell response.

To summarize, regardless of the substrate chemistry, the stimulator identity, matrix coating method (chemical coupling or physical adsorption), or even the type of T cell, all of the studies discussed in this section indicate that T cells are mechanosensitive (Table I). While a broad range of mechanical stiffness has been probed, most of the work to date has focused on use of culture systems that are much stiffer than that of LNs where naïve T cells get activated *in vivo*. The fact that trends in the results are not necessarily consistent highlights the need for experimental designs with fully controlled and quantified parameters. This will become increasingly important as this nascent field begins to turn its attention to elucidating the specific mechanisms of how mechanical cues, like substrate stiffness, are translated into chemical signals inducing activation. To address this limitation, it may be necessary to incorporate new types of biomaterials in which the stiffness, ligand density, and viscoelasticity can be independently tuned.

**TABLE I.** Comparison of experimental methods and results of T cell activation in 2D cultures with varying stiffness. Experimental methods and results are not necessarily consistent between studies. There is variability between (from left to right): T cell identity; density of T cells, which is known to impact T cell fate;^55^ substrate chemistry; method of stimulatory ligand presentation; substrate stiffness range; T cell function(s) measured; and reported response outcomes.

Cell type	Seeded cell density (cells/cm^2^)	Substrate chemistry	Activators	Stiffness range (E)	T cell function measured	Activation outcomes	References
Mouse naive CD4^+^	∼3.1 × 10^5^	PAA-gels containing streptavidin	Biotinylated anti-CD3, anti-CD28	∼10–200 kPa	IL-2 production, phosphorylation of SFK and Zap 70	*No change in cell response below 10 kPa *Activation increased with stiffness	Judokusumo *et al.*
Human naive CD4^+^ and CD8^+^	0.8–1 × 10^6^ cell/ml *Culture plate is not specified	PDMS	Physical adsorption of anti-CD3, anti-CD28	100 kPa–2 MPa	IL-2 and IFN-γ production, cell proliferation	*Activation decreased with stiffness	O'Connor *et al.*
Jurkat (immortalized cell line)	Not reported	PAA treated with hydrazine hydrate	Only anti-CD3	1, 5 kPa	Phosphorylation of Zap70, Lat, and SLP76	* Early signaling enhanced with stiffness *Late signaling decreased with stiffness	Hui *et al.*
Mouse CD8^+^	∼3.1 × 10^4^	PAA coated with adsorbed fibronectin	pMHC+ APC (B16 melanoma cells)+ peptide antigen	12, 50 kPa	Target cell killing	CD8^+^-mediated killing increased with stiffness	Basu *et al.*
Human primary CD4^+^	∼5.7 × 10^5^	PAA-gels containing streptavidin	Biotinylated ICAM-1, biotinylated anti-CD3 and anti-CD28	0.5, 6.4, 100 kPa	Migration, morphology, metabolism, cell-cycle-related genes, IFN-γ and TFN-α production, CD25 and CD69 surface marker expression	*Generally activation increased with stiffness. *Some functions had a low stiffness threshold for activation (e.g., cytokine signaling) *Some functions were sensitive to higher stiffness regimes (e.g., metabolism and cell-cycle genes)	Saitakis *et al.*

### C. Emerging methods to explore T cell mechanobiology using 2.5D substrates

**FIG. 5.**
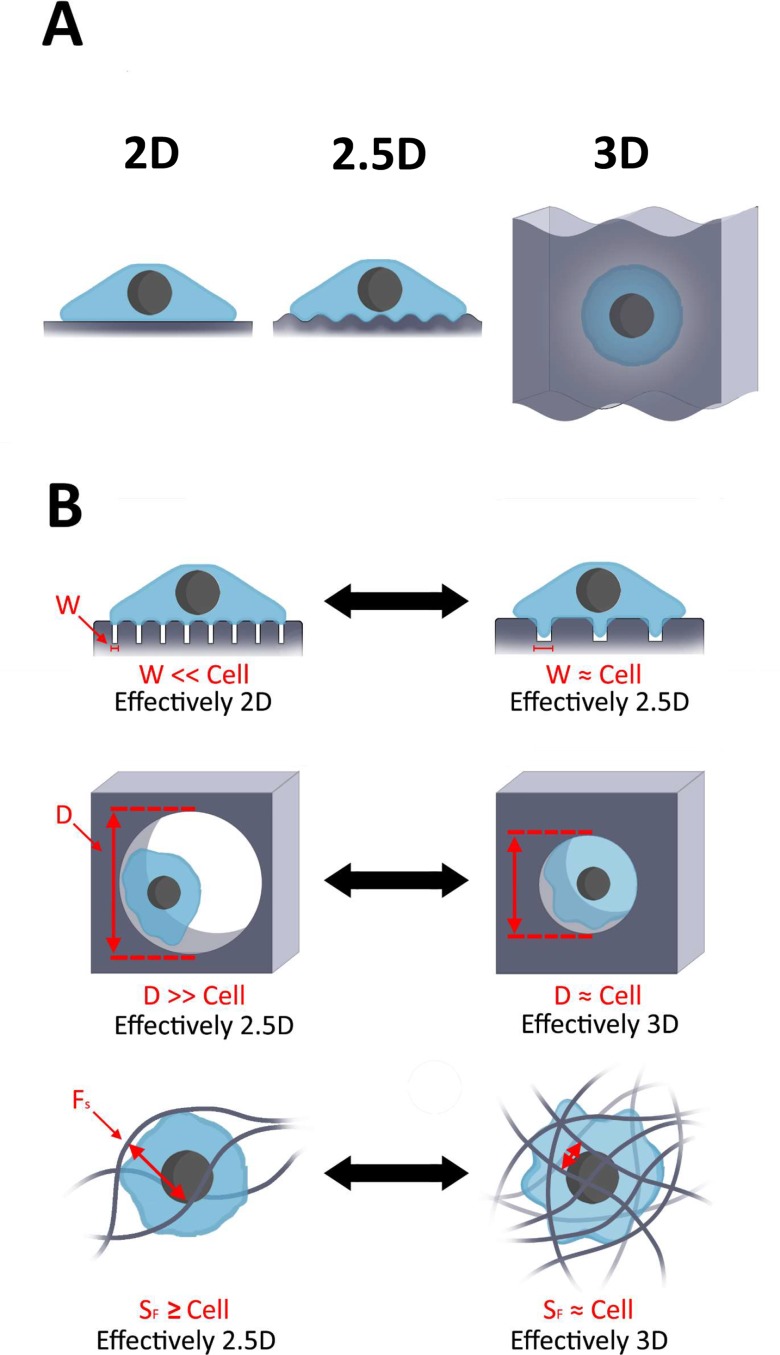
Classification and ambiguity of 2D, so-called 2.5D, and 3D culture systems. (a) Common examples of dimensionality in cell culture systems. Illustration of common 2D, 2.5D, and 3D culture systems. 2D culture systems are those that are perceived by the cell to be flat substrates; so-called 2.5D culture systems have topographical features that induce changes in the membrane curvature of the cell; 3D culture systems include those that completely and continuously encapsulate the cell to provide matrix contact on all sides. (b) Examples of cell culture systems with ambiguous dimensionality. Top panel, micropillar systems could be classified as either 2D or 2.5D, depending on the size and spacing of the pillars with respect to the basal cell membrane geometry. Middle panel, encapsulation systems can be classified as either 2.5D or 3D depending on the pore size diameter (d) of the material. Bottom panel, cell encapsulation within fibrous materials can be classified as either 2.5D or 3D depending on the spacing between fibers (F_S_).

For the purpose of this review, 2.5D substrates are defined as those that have topographical features that can modify the cell membrane curvature but still have one free cell surface, resulting in different apical and basal cell properties. Thus, these systems are distinct from 2D substrates where the basal surface of the cell membrane is typically assumed to be flat. Furthermore, 2.5D systems are distinct from 3D systems where cells are completely encapsulated within a surrounding material [Fig. 5(a)]. By this definition, micropillar systems could be categorized as either 2D or 2.5D, depending on the size and spacing of the pillars and the resulting basal cell membrane geometry [Fig. 5(b)]. Additional materials commonly used that could be considered 2.5D may include topographically varied surfaces, some fibrous or curved substrates, and certain synthetic supported lipid bilayers (SLBs) that induce the cell membrane curvature. T cell studies using these materials have provided insights into the behavior of T cells, including understanding the ramifications of antigen display patterns and measuring the traction forces exerted by T cells. In this section, we will describe these various materials with a focus on the important findings these studies have contributed to T cell biology.

Kwon *et al.* developed nanotopographies of zigzag patterns with varied turning angles and side lengths on polyurethane acrylate surfaces to study the impact of topography on T cell migration.^110^ The patterns were fabricated using UV-assisted capillary force lithography with feature lengths from 15 to 60 *μ*m and turn angles of 45°, 90°, and 135°. The surfaces of the patterned substrates were coated with ICAM-1 to render the materials adhesive for T cells. They observed that sharp turn angles of 45° significantly decreased mean cell velocity, but the feature side lengths had little impact on mobility. Following these findings, the researchers investigated the importance of lamellipodia on motility using the nanopatterned surfaces and an actin-branching inhibitor. Using the Arp2/3 inhibitor CK-636, T cell formation of lamellipodia on the leading cell edge was greatly inhibited. This loss of lamellipodia correlated with a significant decrease in the cell mean velocity, as well as an increase in T cells becoming “trapped” at the turning points. These results demonstrate that lamellipodia are integral for rapid movement of T cells on complicated topographical environments.

Hu *et al.* recently developed a 96-well plate platform termed the Integrated Mechanobiology Platform (IMP) for the high-throughput screening of cellular response to topographical features.^21^ The plate bottoms for this platform are formed by casting PDMS onto molds previously patterned with various topographies via electron-beam lithography. These bottoms are then bonded onto bottomless 96-well plates; thus, each topography is separated into individual wells. This platform enables use of a variety of cell analysis techniques including enzyme-linked immunosorbent assay (ELISA) and flow cytometery, which are not compatible with standard microarray chips. Furthermore, the PDMS-coated well bottoms are also compatible with several microscopy techniques including confocal microscopy. Proof-of-concept studies explored T cell responses to various topographies coated with anti-CD3 and anti-CD28. On grid-patterned topographies, T cell activation (as measured through IL-2 production), spreading, and proliferation were significantly decreased on patterns with 100 nm wide trenches compared to cells on patterns with 200 or 300 nm wide trenches. To demonstrate potential applications for drug screening, they demonstrated that the myosin inhibitor blebbistatin in combination with a 200 nm trench led to an enhancement of IL-2 production. This platform may be useful for quickly identifying topographical patterns that influence T cell biology.

**FIG. 6.**
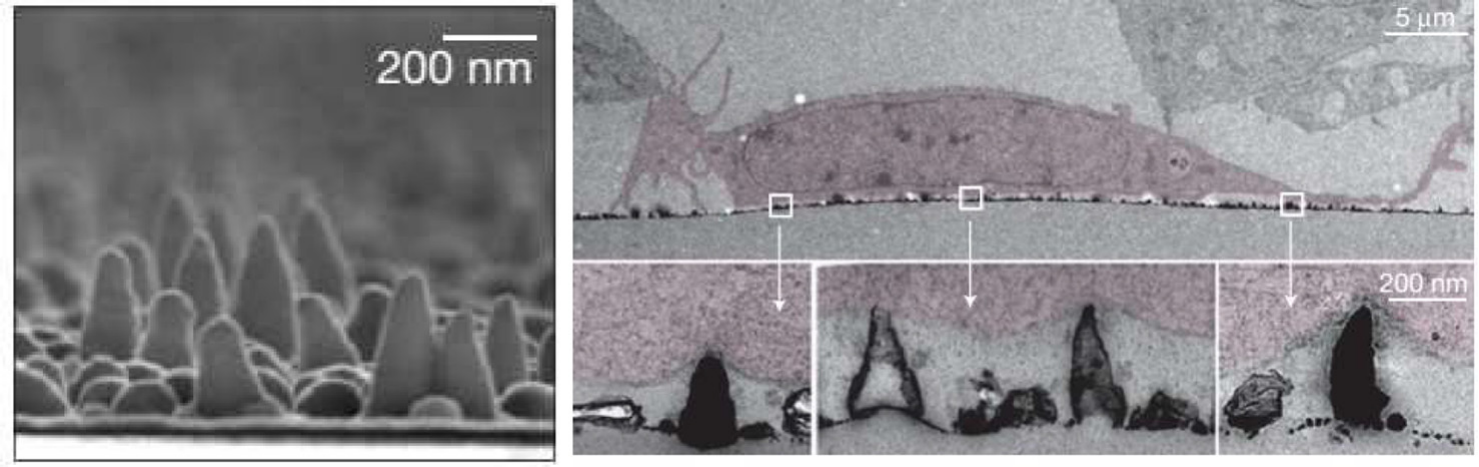
Nanocone platform. Left, scanning electron microscope (SEM) side image of the nanocones. Right, transmission electron microscopy (TEM) image of NIH 3T3 fibroblast cells (colored red), in which the nanocones are inducing a curvature in the plasma membrane of the cells. Reproduced with permission from Galic *et al.*, Nat. Cell Biol. **14**(8), 874 (2012). Copyright 2012 Nature Publishing Group.^112^

Nano and micro technologies enable the control of a myriad of physical features, including topographical geometry, surface roughness, and curvature, which may be key in designing controlled mechanical microenvironments. One area that has not been as widely studied, but is recently becoming of interest, is the link between the membrane curvature and mechanotransduction through the Bin/Amphiphysin/Rvs (BAR) domain proteins, which are found in many types of cells including T cells.^111^ Curvature changes of the lipid membrane are involved in a number of cellular processes, including facilitating filopodia formation and cell division. Similarly, the BAR proteins are also known to play a role in a wide range of cellular processes, such as cortical actin structure regulation and endocytosis. Galic *et al.*, reported that induction of curvature in the plasma membrane of migrating 3T3 cells by nano-cones triggers the recruitment of isolated BAR domains to the sites of the local membrane curvature (Fig. 6).^112^ An alternative technique to alter the local membrane curvature is the use of supported lipid bilayers (SLBs) on coated-curvature-controlled platforms, where different curvature radii are fabricated using colloidal assembly.^113^ SLBs are made of a phospholipid bilayer created through the spontaneous adsorption or fusion of phospholipid vesicles onto the supporting surface. Coating with SLBs is beneficial as it results in a biocompatible surface that enables ligand fluidity as well as controlled composition of stimulatory ligand density and rigidity. SLBs were used as early as 1984 as aAPCs to elicit an immune response from T cells^114^ and have shown great utility in probing T cell activation. Additionally, artificial diffusion barriers can be installed, thus providing “corrals” of bilayers with potentially unique characteristics compared to the rest of the surface. Mossman *et al.* used SLBs with nanometer-scale geometric constraints to frustrate normal IS formation.^115^ In this work, they found that the signaling activity depends on the radial position of T cell receptors. Combining these types of T cell activation studies with measurements of membrane curvature and local signaling of mechanotransduction proteins may yield new insights into how mechanical forces potentiate T cell activation.

### D. Emerging methods to explore T cell mechanobiology in 3D scaffolds

While there is a wealth of knowledge about maintaining cell cultures in 2D, these models are inherently limited when trying to mimic the native 3D architecture. Many cells behave differently in 2D culture systems compared to 3D systems.^116^ Thus, studying T cell mechanobiology in a 3D context may provide insights that otherwise could not, or would not as accurately, be observed. While no studies of T cell mechanotransduction within 3D microenvironments have yet been reported, there have been several elegant strategies developed to incorporate T cells into 3D systems. Since most of these systems use polymeric materials with tunable mechanical properties, these platforms could be feasibly extended into studies of 3D T cell mechanotransduction. Several approaches can be used to study mechanical forces in 3D microenvironments, including particle tracking microscopy in 3D, diffusing wave spectroscopy, and dynamic light scattering (methods reviewed elsewhere^117^).

To date, the study of T cells within 3D engineered platforms has largely focused on the design of synthetic lymphoid organoids (SLOs). The broader goal of this field is to leverage our native immune system as a therapeutic by artificially providing cues that drive the accumulation of adaptive immune cells, e.g., T cells, towards a tumor or site of infection. Since T cells are maintained, activated, and differentiated in the secondary LNs, SLOs are being developed as *in vitro* and *in vivo* systems to better understand what critical features can be leveraged to enhance immunotherapies.^118^ Scaffold materials explored for these applications include both synthetic and isolated native materials including alginate, agarose, polyamide, polyethylene glycol (PEG), and collagen. Typically, these include embedded stromal cells, which are known to secrete various ECM proteins into the surrounding microenvironment. As a result, these culture systems lack precise control over the mechanical cues sensed by the T cells. Nevertheless, these studies demonstrate the feasibility of embedding T cells in an *in vitro* 3D culture system, and several of these platforms may be useful for further investigation of how mechanics affect T cell activation.

**FIG. 7.**
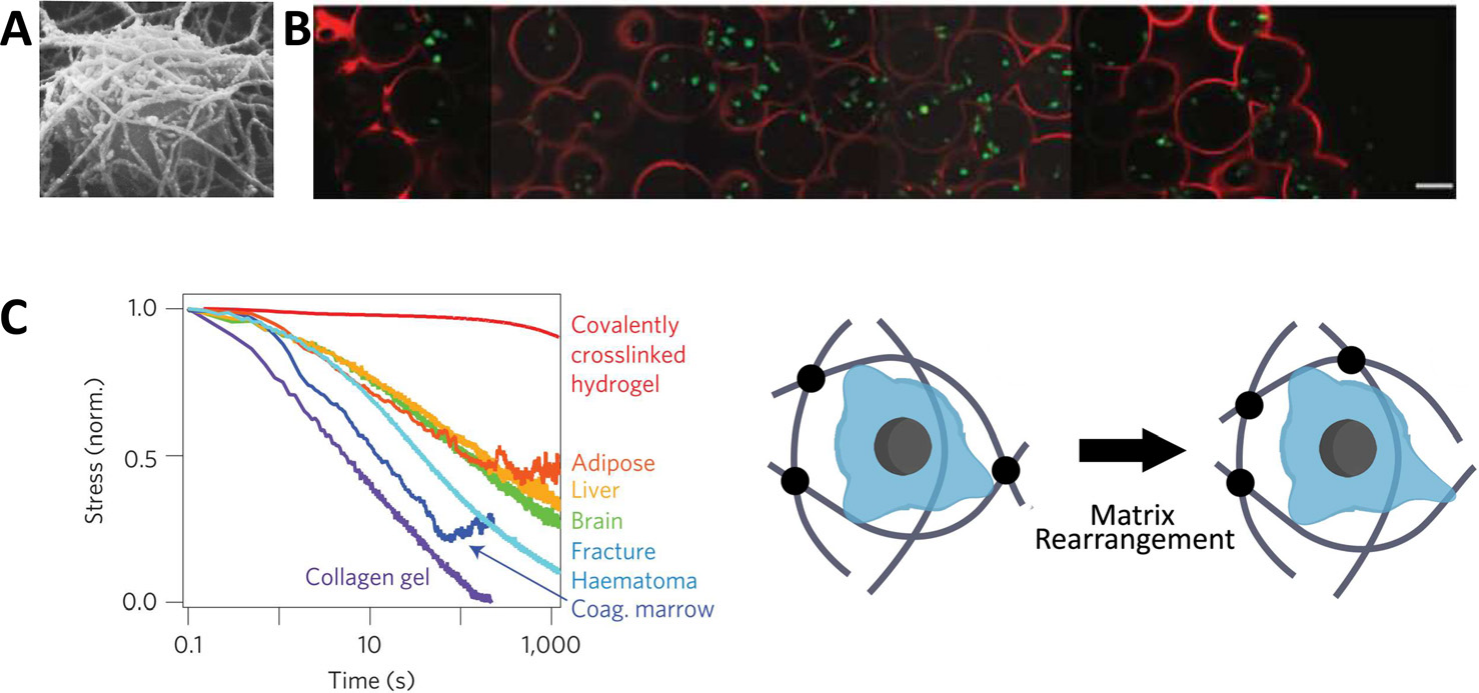
T cells encapsulated within various matrices. (a) T cell in a confined/continuous 3D collagen matrix gel. A scanning electron micrograph of a T cell encapsulated within a collagen gel matrix. Reproduced with permission from Schor *et al.*, J. Cell Biol. **96**(4), 1089 (1983). Copyright 1983 Rockefeller University Press.^119^ (b) T cells encapsulated in an inverted colloidal crystal (ICC) scaffold. Confocal fluorescence microscopy images of thin sections cut through a ∼1 mm-thick ICC scaffold. T cells are labeled with carboxymethylfluorescein diacetate (CMFDA) (green); PEG ICC (red) has 80-*μ*m void diameters. Scale bar: 41 *μ*m. Reproduced with permission from Stachowiak and Irvine, J. Biomed. Mater. Res., Part A **85**(3), 815 (2008). Copyright 2007 Wiley Periodicals, Inc.^120^ (c) Matrix viscoelasticity. The left panel demonstrates that many native tissues are viscoelastic, and thus exhibit time-dependent stress relaxation. Reproduced with permission from Chaudhuri *et al.*, Nat. Mater. **15**(3), 326 (2016). Copyright 2015 Nature Publishing Group.^121^ The right panel schematic shows how stress relaxation can occur at a molecular level due to rearrangements of the polymer network crosslinks over time.

Schor *et al.*, was the first to encapsulate naïve T cells in a 3D confined/continuous hydrogel [Fig. 7(a)].^119^ In their studies, collagen matrices were used to study the kinetics of lymphocyte penetration and to determine the T cell distribution throughout the collagen matrix. This matrix was specifically selected as it is a constituent of the tissue stroma through which lymphocytes migrate *in vivo*. Lymphocytes were observed to migrate in a “random walk” fashion, and lymphocytes seeded onto the surface of the collagen gel were observed to penetrate into the gel matrix. This surprising observation of cell infiltration into the collagen gel did not appear to involve collagenolytic activity and is hypothesized to be a consequence of the uniquely small size (7 *μ*m) and fast speed (10 *μ*m/min) of T cells. Gunzer *et al.* expanded upon this paper and used DCs to activate naïve T cells in a 3D collagen gel.^14^ This work provided the first indication that T cell activation can occur in a 3D *in vitro* model and provided insights into a T cell's migration pattern after encountering antigen-loaded DCs. Both papers used physically crosslinked collagen and observed T cell migration rates similar to *in vivo* rates of 10–11 *μ*m/min.

Several other biopolymers are present in the T cell's native microenvironment, including hyaluronan and laminin. However, use of naturally derived ECM polymers in *in vitro* studies have the limitations of batch-to-batch variability, undergoing microarchitectural remodeling through protease degradation, and having a limited range of tunability in terms of mechanical and biochemical properties. Recently, a variety of naturally derived ECM polymers and synthetic polymers have been chemically modified to create semisynthetic hydrogel systems that address many of these limitations.^122^ Due to their control over matrix mechanical properties, these materials may be well-suited for future studies of T cell mechanotransduction in 3D. However, since most synthetic polymer scaffolds typically have pore sizes that are much smaller than those found in natural-polymer scaffolds, typically cells seeded onto the top surface of these gels will not be able to infiltrate into the scaffold. Thus, the cells are usually pre-mixed with the gel precursors in order to attain high levels of homogeneous cell-loading. This fabrication process can expose the cells to potential damage from crosslinking reagents or transient exposure to non-physiological environmental conditions, such as low pH or temperature, to induce gelation of the scaffold. In addition, the small pore sizes in these synthetic systems typically require that the scaffold must degrade or remodel over time in order to enable migration of encapsulated cells.^123^

An alternative approach to promote cell migration into 3D scaffolds is the use of inverted colloidal crystals (ICCs), also referred to as inverse opal hydrogels [Fig. 7(b)]. In the ICC method, a sacrificial porogen is used to create a macro-porous scaffold; thereby providing an isotropic 3D environment, with long range order, uniform interconnectivity, and tunable pore size, typically within the range of 20–500 *μ*m. The ICC structure can achieve a high porosity of ∼74%, which facilitates oxygen and nutrient diffusion and the homogeneous distribution of cells throughout the scaffold.^124,125^ T cells have been reported to be sensitive to oxygen transport within 3D collagen gels, thus, this is another important consideration when designing a 3D platform for study of T cells.^126^ Macro-porous systems may be categorized as either 2.5D or 3D platforms depending on the pore size. When the scaffold pore size is much larger than the dimension of a cell, the cell effectively encounters a 2.5D local environment and can utilize the associated void space to efficiently migrate [Fig. 5(b)]. In contrast, if the pore size is about the size of a single cell or cell cluster, then the cell may become entrapped within an effectively 3D local environment [Fig. 5(b)]. Stachowiak and Irvine *et al.* developed a composite of an ICC-patterned PEG hydrogel infused with fibrillar collagen as a lymphoid tissue model to study immune cell migration.^120,127^ The highly motile T cell migratory patterns observed within this scaffold were similar to those found in native secondary lymphoid organs. Because a wide range of different materials with tunable mechanical properties could be used to fabricate ICC scaffolds, this technique may be an attractive option to begin moving T cell mechanotransduction studies into 3D.^125^

So far, only methods for T cell encapsulation *in vitro* have been reviewed. Bridging to the *in vivo* setting, Monette *et al.* recently demonstrated that T cells can be encapsulated in a thermogel and then injected into a rat to allow for localized delivery.^128^ Specifically, the goal was to deliver tumor-expanded CD8^+^ T cells to a tumor mass for cancer immunotherapy. The injectable and biodegradable scaffold is chitosan-based, and it allows for T cell growth, proliferation, and survival *in vitro* as well as *in vivo* for periods of up to 3 weeks.^129^ Its mechanical properties are tunable and can reach more physiologically relevant levels (0.1–5 kPa), thus it can effectively mimic the stiffness experienced by cells within the LNs and other soft tissues. These results together with the fact that thermogels have been used to study mechanotransduction in other cell types,^130^ indicate that this chitosan-based thermogel formulation may be promising in studying 3D T cell mechanobiology.

When designing a material platform to mimic the mechanical properties of native tissue, it may also be critical to consider the time-dependent mechanics of the ECM. Native tissues are viscoelastic, i.e., they possess both time-independent elastic stiffness and time-dependent viscosity, which enables the matrix to undergo molecular-level remodeling and stress relaxation over time [Fig. 7(c)]. However, most *in vitro* studies of cell mechanobiology to date have focused only on the cellular effects of elastic stiffness. Recent work by Chaudhuri *et al.* and Cooper-White *et al.* have demonstrated that cells can be exquisitely sensitive to the time-dependent mechanical behavior of the matrix.^121,131^ These effects are thought to be due to local clustering of cell-adhesive ligands that can occur more readily on matrices that have faster rates of stress relaxation. It remains to be seen if similar mechanisms may be important for T cells, which are relatively low adherent compared to many other cell types.^132^

To summarize, there is a significant trade-off between designing a 3D system that accurately mimics the physiological complexity of the native microenvironment and yet is simplified enough to maintain a feasible level of reproducibility. The study of 3D T cell mechanobiology is nascent, and there are many experimental variables that need to be explored in order to conduct a reliable and biologically relevant experiment.

### E. Emerging methods to explore T cell mechanobiology *ex vivo* and *in vivo:* Lysyl oxidase (LOX) and β-aminopropionitrile (βAPN)

**FIG. 8.**
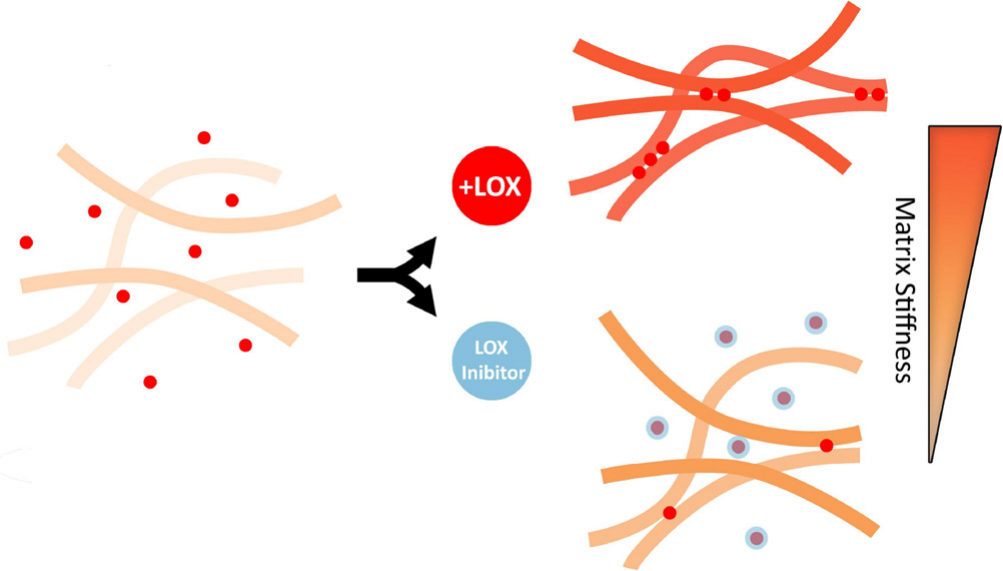
Potential strategy to study mechanotransduction *in vivo* using lysyl oxidase (LOX). Left, schematic of collagen fibrils (beige) with a typical, basal-level of lysyl oxidase (LOX, red). Top right, increased levels of LOX lead to increased covalent crosslinking of adjacent collagen fibers, leading to matrix stiffening. Bottom right, depletion or inhibition of LOX disrupts collagen crosslinking and prevents stiffening.

To date, research in T cell mechanotransduction has been exclusively performed *in vitro.* However, to gain insights that are truly physiologically relevant, it may be necessary to develop experimental protocols that work with *in vivo* or *ex vivo* systems. Here we discuss one potential opportunity to pursue these studies using the lysyl oxidase (LOX) family of proteins (Fig. 8). Many diseases that are linked to ECM mechanical changes have also been shown to have abnormal levels of LOX. While most healthy tissues have a low level of LOX expression (which is secreted by fibroblasts, smooth muscle cells, osteoblasts, and vascular endothelium), various diseases including cancer are associated with either upregulation or downregulation of LOX.^133,134^

LOX is an enzyme family that crosslinks collagen and elastin through oxidative deamination of lysine residues to form a semialdehyde, which subsequently enables covalent crosslinking to adjacent collagen fibers and ECM stiffening.^134–136^ On the other hand, inhibition of LOX, which can be achieved using blocking antibodies, specific RNA interference, or β-aminopropionitrile (βAPN), disrupts this stiffening.^137^ βAPN is a small, irreversible, competitive inhibitor that targets the catalytic domain of LOX.^138^ Importantly, inhibition of LOX with βAPN *in vivo* can alter the matrix mechanics without changing the matrix composition, fiber density, and fiber organization, opening the door to mechanistic studies.^137^ Manipulation of LOX activity *in vivo* and *ex vivo* has been used to probe the role of tissue mechanics in the progression of cancer.^135^ For example, Levental *et al.* demonstrated that upregulation of LOX resulted in stiffer tissue and greater tumor invasion, while inhibiting LOX resulted in more compliant tissue and reduction in metastatic spread. Furthermore, LOX upregulation correlated with increased integrin clustering and subsequent enhancement of growth factor signaling. As CD8^+^ T cells have been implicated in cancer progression, one interesting avenue may be to evaluate the effects of this LOX/βAPN treatment on local T cell responses.

## V. CONCLUSION

Through the course of this review, we have highlighted some of the key studies that provide the foundation for understanding T cell mechanobiology, with a focus on the engineering techniques that enabled these studies. Continuing to improve our understanding of T cell mechanobiology will require close collaborations between researchers with both engineering and immunology backgrounds. As has been pointed out throughout this review, in many instances, the techniques for more sophisticated mechanobiology experiments have been demonstrated already with other cell types, but are yet to be applied to questions fundamental to T cell biology. In other instances, new engineering techniques may need to be developed to both quantitatively perturb and measure mechanical forces imposed on, generated by, and found within T cells. Importantly, attention to using materials and forces that recapitulate physiological conditions will be vital to understanding T cell behaviors in both healthy and diseased tissues.

Improved understanding of T cell mechanobiology will be valuable for both fundamental and applied immunology. Although currently underappreciated, the ability of mechanical forces to dramatically alter T cell activation may be exploited in the future to control T cell behavior in a variety of immunotherapies. These might include enhancement of T cell activation to better fight off infection or suppression of T cell activation to better regulate autoimmunity. In addition, as engineered T cell therapies become more prevalent, T cell mechanobiology may also offer new opportunities to efficiently expand and program T cells *ex vivo*. Thus, by merging the disciplines of bioengineering, mechanobiology, and T cell immunology, new fundamental and translational discoveries will be made.
